# Electroconductive and Anisotropic Structural Color Hydrogels for Visual Heart‐on‐a‐Chip Construction

**DOI:** 10.1002/advs.202105777

**Published:** 2022-03-28

**Authors:** Lingyu Sun, Zhuoyue Chen, Dongyu Xu, Yuanjin Zhao

**Affiliations:** ^1^ Department of Rheumatology and Immunology Institute of Translational Medicine The Affiliated Drum Tower Hospital of Nanjing University Medical School Nanjing 210008 China; ^2^ State Key Laboratory of Bioelectronics School of Biological Science and Medical Engineering Southeast University Nanjing 210096 China; ^3^ Oujiang Laboratory (Zhejiang Lab for Regenerative Medicine, Vision and Brain Health) Wenzhou Institute University of Chinese Academy of Sciences Wenzhou Zhejiang 325001 China; ^4^ Institute for Stem Cell and Regeneration Chinese Academy of Science Beijing 100101 China

**Keywords:** carbon nanotube, heart‐on‐a‐chip, hydrogel, microfluidics, structural color

## Abstract

Heart‐on‐a‐chip plays an important role in revealing the biological mechanism and developing new drugs for cardiomyopathy. Tremendous efforts have been devoted to developing heart‐on‐a‐chip systems featuring simplified fabrication, accurate imitation and microphysiological visuality. In this paper, the authors present a novel electroconductive and anisotropic structural color hydrogel by simply polymerizing non‐close‐packed colloidal arrays on super aligned carbon nanotube sheets (SACNTs) for visualized and accurate heart‐on‐a‐chip construction. The generated anisotropic hydrogel consists of a colloidal array‐locked hydrogel layer with brilliant structural color on one surface and a conductive methacrylated gelatin (GelMA)/SACNTs film on the other surface. It is demonstrated that the anisotropic morphology of the SACNTs could effectively induce the alignment of cardiomyocytes, and the conductivity of SACNTs could contribute to the synchronous beating of cardiomyocytes. Such consistent beating rhythm caused the deformation of the hydrogel substrates and dynamic shifts in structural color and reflection spectra of the whole hybrid hydrogels. More attractively, with the integration of such cardiomyocyte‐driven living structural color hydrogels and microfluidics, a visualized heart‐on‐a‐chip system with more consistent beating frequency has been established for dynamic cardiomyocyte sensing and drug screening. The results indicate that the electroconductive and anisotropic structural color hydrogels are potential for various biomedical applications.

## Graphical abstract

An electroconductive and anisotropic structural color hydrogel by simply polymerizing non‐close‐packed colloidal arrays on super aligned carbon nanotube sheets (SACNTs) is presented. With the integration of such cardiomyocyte‐driven living structural color hydrogels and microfluidics, a visualized heart‐on‐a‐chip system with more consistent beating frequency has been established for dynamic cardiomyocyte sensing and drug screening.

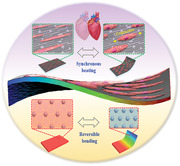

## 1 Introduction

Organ‐on‐a‐chip, constructed based on a microfluidic system to mimic the physiology and functionality of human organs, has been regarded as a novel approach to facilitate a paradigm shift in drug development, disease modeling, and biological mechanism research.^[^ ^1^, ^2^, ^3^, ^4^, ^5^, ^6^, ^7^, ^8^ ^]^ As an important branch of organ‐on‐a‐chip, heart‐on‐a‐chip has exhibited superiorities in creating simulated structures, providing fluidic shear, constructing cardiac disease models, and researching cytophysiological variation, and has been employed in drug‐cardiotoxicity evaluation, pathological mechanism, individualized precision medicine, etc.^[^ ^9^, ^10^, ^11^, ^12^, ^13^, ^14^ ^]^ Among various designs of heart‐on‐a‐chip, the one based on patterned structural color hydrogels has attracted interest in recent years.^[^ ^15^, ^16^, ^17^, ^18^ ^]^ Taking advantage of the features of these colorful hydrogels, the cardiomyocytes could be aligned and their beating would be reflected on the substrates bending or drawing, both of which could self‐report an obvious periodic color variation of the materials for the microphysiological visuality in the heart‐on‐a‐chip.^[^ ^19^, ^20^, ^21^ ^]^ Although with many progresses, the current construction of the patterned structural color materials‐based heart‐on‐a‐chip consists of several steps, including assembling, replicating, and chemical etching. Such necessary steps result in complexity in the manufacture, and residual etching liquid may leave biocompatible issues. In addition, due to the lack of electrical conductivity of these structural color hydrogels, the beating inconsistency of the cardiomyocytes in the heart‐on‐a‐chip was usually unavoidable. Therefore, a simple and fast method to construct electroconductive and patterned structural color substrates for heart‐on‐a‐chip is still anticipated.

**Figure 1:**
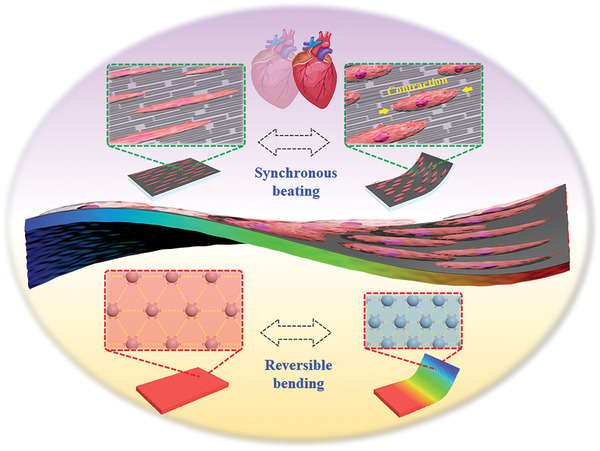
Schematic diagram showing the described anisotropic hydrogel consisting of a colloidal array‐locked hydrogel layer with brilliant structural color on one surface and a conductive methacrylated gelatin (GelMA)/SACNTs film on the other surface.

In this paper, we presented a novel electroconductive and anisotropic structural color hydrogel by simply polymerizing non‐close‐packed colloidal arrays on super aligned carbon nanotube sheets (SACNTs) for visualized and accurate heart‐on‐a‐chip construction, as schemed in **Figure** **1**. Non‐close‐packed colloidal arrays are a typical class of photonic crystals with photonic band gaps (PBGs) to manipulate photons propagation.^[^ ^22^, ^23^, ^24^, ^25^ ^]^ Different from the close‐packed ones, non‐close‐packed colloidal arrays, which could be simply derived from the polymerization of hydrogel monomers dispersed with charged colloidal nanoparticles, have superiority in implementing flexible bending or volume changes without harmful chemical etching process and other complex treatments.^[^ ^26^, ^27^, ^28^, ^29^, ^30^, ^31^, ^32^, ^33^ ^]^ Thus, they are more valuable in sensing‐related applications, such as optical devices, thermal sensing, and biochemical detection. In contrast, SACNTs are extensively investigated in electronics, energy storage, catalysis, etc., attributed to their extraordinary mechanical performance, electrical conductivity, and high flexibility.^[^ ^34^, ^35^, ^36^, ^37^ ^]^ Particularly, the anisotropic structure of SACNTs imparts them with the capacity of cellular alignment, thus expanding their practical value in the biomedical field.^[^ ^38^, ^39^, ^40^ ^]^ Therefore, it is conceivable that the combination of non‐close‐packed colloidal arrays and SACNTs would create a new conductive substrate with the anisotropic structure for heart‐on‐a‐chip construction in a facile manner.

Herein, we fabricated the desired electroconductive and anisotropic structural color hydrogels by coating and polymerizing colloidal array‐dispersed pregel solution on the surface of methacrylated gelatin (GelMA)/SACNTs film. With the gelation of acrylamide pregel, the colloidal arrays were permanently locked in the hydrogel network, thus displaying a brilliant structural color as that of the pregel solution. Meanwhile, the SACNTs distributed on the other surface of the hybrid hydrogels maintained their ordered arrangement and electroconductivity without being disturbed in the polymerization process. It was found that the anisotropic SACNTs in the hybrid hydrogels could effectively induce the adhesion and alignment of cardiomyocytes to form a bionic structure, and that the conductivity of the SACNTs contributed to the synchronous beating rhythm of the cardiomyocytes on the hydrogels. Such consistent beating rhythm would cause the deformation of the hydrogel substrates, companying with dynamic shifts in structural colors and reflection spectra of the hybrid hydrogels. More attractively, by integrating the cardiomyocytes cultured living structural color hydrogels into a mixed microfluidic device, a visualized heart‐on‐a‐chip system with a more consistent beating frequency could be established. Based on this system, we have demonstrated its practical value for cardiomyocyte sensing and drug evaluation. These features implied that the electroconductive and anisotropic structural color hydrogels and their derived organ‐on‐a‐chip are ideal candidates in drug development and further biomedical regulation applications.

## 2 Results and Discussion

In a typical experiment, the SACNTs integrated conductive structural color hydrogels were fabricated through a simple polymerization strategy. During the preparing process, the surface of silica nanoparticles decorated with negative charge was first prepared and mixed in the acrylamide (AAm) pregel. Under the effect of charge repulsion and minimum energy configuration, the silica nanoparticles would assemble into non‐close‐packed arrays. Due to the ordered arrangement of nanoparticles, the silica nanoparticle‐doped imparted the structural color hydrogel with PBGs properties and characteristic reflection wavelengths, which could be calculated according to Bragg's equation:

(1) $$\lambda \;=\;2d{\left({n_{average}}^{2}-cos^{2}\theta \right)}^{\frac{1}{2}}$$

where *λ* refers to the characteristic wavelength, *d* is the lattice spacing within silica arrays, *n_average_* symbolized for the mean refractive index of the composite hydrogel, and *θ* represents the glancing angle, as known as the angle within the incident light and the hydrogel plane. According to this equation, it could be found that the position of reflection peak could be regulated via lattice spacing *d*, the hydrogel constitution, and glancing angle *θ*. When the hydrogel component and glancing angle are fixed, the reflection peak only depends on *d* that is decided by particle size and inter‐particle spacing, which could be adjusted by using different sizes of silica particles or tuning the concentrations of the charged silica arrays. When the concentration of surface‐charged silica nanoparticles achieved a certain range, the hybrid pregel solution would display brilliant structural colors in a continuously adjustable manner.

**Figure 2:**
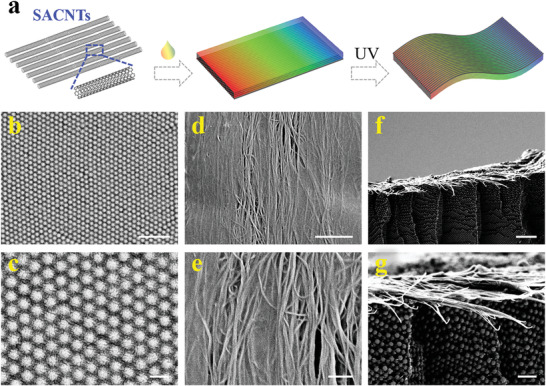
a) Schematic diagram showing the fabrication of anisotropic and conductive structural color hydrogel. b–e) Scanning electron microscopy (SEM) images of the b,c) non‐close‐packed silica nanoparticles and d,e) GelMA/SACNTs, respectively. f,g) Side view showing the interface microstructure of SACNT‐integrated structural color hydrogel. Scales bars are 1 µm in (b), 200 nm in (c), 2 µm in (d) and (f), and 500 nm in (e) and (g).

To achieve the free‐standing electroconductive structural color hydrogels, the SACNTs that aligned on glasses were first infiltrated by biocompatible GelMA solution for pre‐polymerization and then coated with the AAm pregel solution dispersed with charged silica nanoparticles for ultraviolet (UV) irradiation (**Figure** **2**a). Because of the prepolymerization of GelMA and the subsequent polymerization of Aam hydrogel, the assembly of silica nanoparticles would not be affected, and the non‐close‐packed arrays were permanently locked in the hydrogel network (Figure 2b,c), thus ensuring the optical performance of the structural color hydrogel. In this case, the structural color hydrogel was composed of a colloidal array layer on one surface and a SACNTs layer on the other surface. It could be found that the SACNTs maintained their orientation and arrangement after polymerization (Figure 2d,e). Despite the inevitable deformation in sample preparation for observation, the non‐close‐packed silica particles could arrange in ordered arrays with intervals between adjacent particles, together with a SACNTs layer that distributed on their surface, even from the cross‐section view (Figure 2f,g). These observed structures guaranteed the functional features to be applied in the further heart‐on‐chips and their applications.

When the hydrogels were polymerized, the structural colors were closely related to the distance among nanoparticles, which depended on the swelling degree of the hydrogel network. To obtain stable optical properties, the hydrogel constituent was investigated to minimize the swelling ratio. During the process, the optical features of structural color hydrogels with different concentrations of polyethylene glycol diacrylate (PEGDA) additives were observed at the state of pregel solution and polymerized hydrogel in water. The wavelength variation under these two conditions reflected the swelling degree of the hydrogel, where the greater differentials indicated the greater swelling. It was found that the addition of PEGDA could effectively inhibit the swelling of AAm networks and the derived red‐shift in characteristic wavelength. When the ratio of the hydrogel of AAm and PEGDA reached 15:3, it would lead to a small wavelength shift by hydrogel swelling (Figure S1, Supporting Information). As the proportion of PEGDA further increased, the red‐shift tendency decreased close to zero, while the modulus of hydrogels would correspondingly increase along with the addition of PEGDA concentration. To investigate the impact in hydrogel film's flexibility, the relationship between stress and bending angle of the hybrid hydrogel and the hydrogel with silica arrays were respectively tested. It was found that the stress of the hybrid hydrogel increased with the increase of PEGDA additive, whose tendency was similar to the hydrogel with silica arrays (Figure S2, Supporting Information). Considering both the optical and mechanical features, the proportion of AAm and PEGDA at 15:3 was chosen for further experiments.

**Figure 3:**
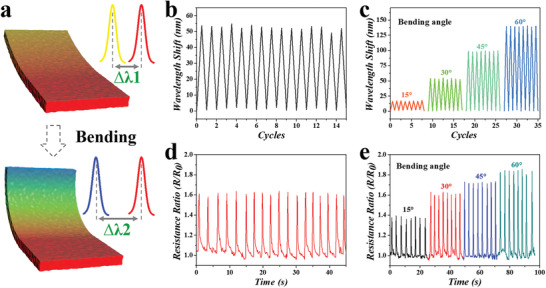
a) Schematic illustration of the structural color variation with different bending degrees. b) Representative optical stability test of the electroconductive structural color hydrogel within bending cycles (30°). c) Cycled tests of the wavelength shift under different bending degrees. d) Electrical stability test of the electroconductive structural color hydrogel within bending cycles (30°). e) Cycled tests of the relative resistance change under different bending degrees.

As the silica nanoparticles were arranged in non‐close‐packed arrays, the structural color hydrogel substrate followed Bragg's equation and possessed the angle‐dependent optical features. According to Equation (1), as the glancing angle *θ* decreased, the increase of the incident angle would lead to the blue‐shift of characteristic wavelengths. When the observing position was fixed vertical to the plane in which the hydrogel was without bending, the observed optical wavelength of the hydrogel would blue‐shift with hydrogel bending (**Figure** **3**a). Based on this, a series of cycle tests were utilized to confirm the optical stability of the structural color hydrogel substrates. Taking the 30° bending as an example, the hydrogel showed about 53 nm blue‐shifts in the repeating bending cycles, without evident sharp increase or decrease in the process (Figure 3b). Additionally, the variable bending angle cycle tests indicated the relationship between optical features and the bending angles. It was demonstrated that the optical shifts increased along with the bending angle increase (Figure 3c). There were few observed variations of the optical signals in the repeating bending cycles, which demonstrated the stability of the structural color hydrogel substrate. Noted that multiple independent substrates showed similar results and thus proved their reproducibility (Figure S3, Supporting Information). The detailed wavelength‐bending angle relationship was tested and described (Figure S4, Supporting Information). These results indicated that the structural color substrate was suitable for optical sensing applications.

In addition to optical functions, the electrical properties of the SACNTs‐integrated structural color were also investigated by a series of electrical signal‐bending angle tests. It was found that the resistance of the hydrogel would change with bending and exhibited a stable variation range in bending cycles (Figure 3d). Similar to the optical features, the electrical resistance of the structural color hydrogel substrate also showed bending angle‐dependence that increased with the bending angles (Figure 3e). Taking advantage of the angle‐dependent optical and electrical properties, the relationship between them could be concluded. As the hybrid hydrogel proportion and silica concentration fixed, the optical and electrical features depended on the bending angle, in which the characteristic wavelength decreased, and resistance ratio grew with the increasing bending angle (Figure S3b and S4b, Supporting Information). In addition, the angle‐dependent features possessed the fast‐response ability. By adjusting the bending cycles at different frequencies of 0.5, 1, 1, 5, and 2 Hz, which was the potential cardiomyocyte beating rate under normal culture and drug stimulation, the hydrogel substrate exhibited a quick response to the bending with stability and consistency (Figure S5, Supporting Information). These features implied that such structural color hydrogel substrates were of great value in sensing part of heart‐on‐a‐chip.

**Figure 4:**
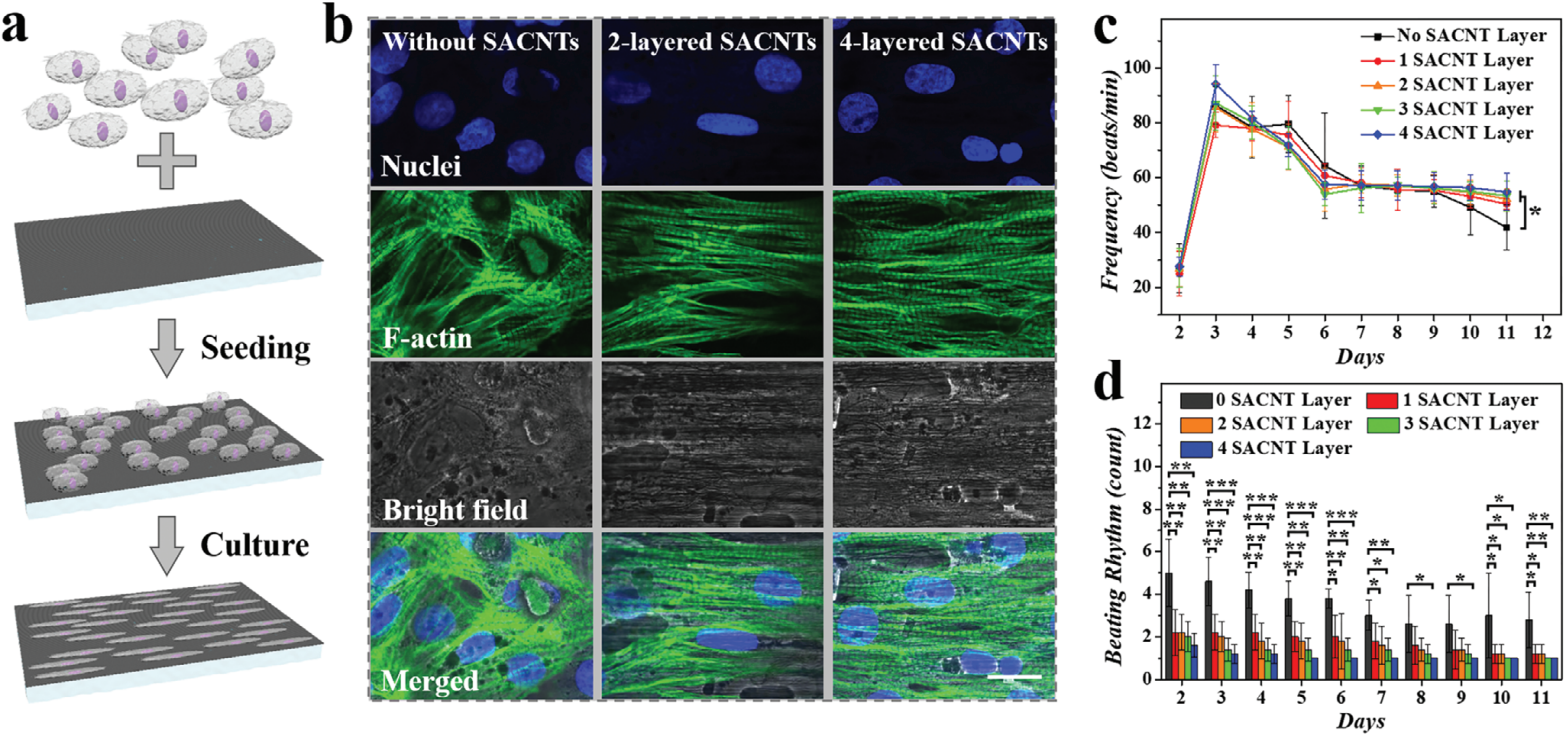
a) Schematic illustration of the culture and alignment of cardiomyocytes on the electroconductive structural color hydrogel. b) Fluorescent images (nuclei in blue and F‐actin in green) showing the morphology of cardiomyocytes cultured on substrates with/without SACNTs. c) Cellular frequency statistics on substrates integrated with different layers of SACNTs. d) Statistic diagram showing the pacemaker numbers on different substrates. Scale bar is 25 µm. Error bars represent standard deviation (SDs) and sample size (*n*) was 3. *p < 0.05, **p < 0.01, ***p < 0.001 when compared with the group with no SACNT layer.

To further investigate the cardiac growth and beating behavior, the GelMA/SACNTs side of electroconductive structural color hydrogel was placed upward and then seeded with neonatal rat cardiomyocytes. Several structural color hydrogels with different configurations were utilized as substrates for cardiomyocytes culture to verify the biocompatibility and cellular adhesion of these materials. It could be found that the biocompatibility was similar among these materials, while the integration of SACNTs and covering of GelMA greatly improved the cell adhesion rate of the substrate (Figure S6, Supporting Information). After confirming the cardiac compatibility and adhesion, the cardiomyocytes were cultured on the GelMA‐decorated electroconductive structural color hydrogel. As the SACNTs possessed excellent orientation morphology and electrical conductivity, different SACNTs layers were integrated on the hydrogel to investigate their effects on cardiomyocytes. According to the F‐actin and nuclei staining results, the existence of SACNTs greatly improved the cellular arrangement along with the direction of SACNTs (**Figure** **4**a,b; Figure S7, Supporting Information). The hydrogel with one‐layer‐SACNT‐integration exhibited striking cell orientation, and the orientation consistency seemed to improve with the increase of the SACNTs layers (Figure S8, Supporting Information), which may be attributed to the increased exposure degree of embedded SACNTs in the hydrogel.

Apart from cellular orientation induction, the effect of SACNTs on the periodic contraction of cardiomyocytes was observed and counted. The SACNTs integration would be conducive to the cardiomyocytes maintaining regular contraction cycles, especially after a week culture period (Figure 4c). It was worthy of mentioning that the SACNTs could unify the beating rhythm of the cardiomyocytes. The isolated cardiomyocytes on GelMA‐covered structural color hydrogel substrate without SACNT integration existed at several points at different beating rhythms that tended to decrease with the culture time, which may be attributed to the fusion of the cell layer. In contrast to the group without SACNTs, the cardiomyocytes on the GelMA‐infiltrated SACNTs integrated structural color hydrogels exhibited regulated beating rhythm. The beating points on the substrate decreased evidently with the increase of the SACNT layers, especially the four‐layer SACNTs integration led to the greatly unified beating rhythm (Figure 4d; Movie S1, Supporting Information). This impact could be ascribed to the conductivity improvement with the increasing SACNT layer, which benefited the action potential spreading from the first‐contraction cell to other cardiomyocytes. In view of the cell induction effect and the conductivity, the four‐layer SACNTs integrated hydrogels were employed in the subsequent heart‐on‐a‐chip construction.

**Figure 5:**
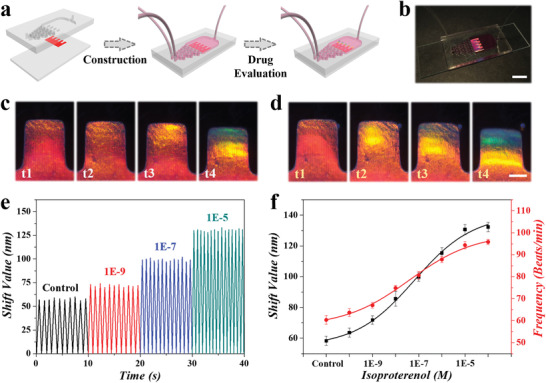
a) Schematic diagram of the fabrication and assembly of heart‐on‐a‐chip for drug evaluation. b) Optical photograph showing the derived heart‐on‐a‐chip. Scale bar is 1 cm. c,d) Microscopy photographs showing the structural color variation in the functional hydrogel c) without or d) with isoproterenol stimulation. Scale bar is 0.5 mm. e) Statistic graph showing the relationship between shift values and beating velocity under different isoproterenol concentrations. f) Relationship revealing the shift values of wavelength and beating frequency change under different concentrations of isoproterenol. Error bars represent SDs (*n* = 5).

As the central visualized sensing component, the electroconductive structural color hydrogel was integrated with a polydimethylsiloxane (PDMS) microfluidics platform and a glass slide to fabricate the desired heart‐on‐a‐chip. The PDMS channel and chamber were obtained by template molding method and treated with plasma, followed by the fixation of structural color hydrogel using the micro‐pillars inside the chamber (**Figure** **5**a,b). Due to the transparency and photopermeability, the PDMS would ensure the optical signal propagation and reception during the sensing process. When the cardiomyocytes in the heart‐on‐a‐chip recovered their autonomous contraction cycles, the inside structural color hydrogel would be driven by cardiac beating and occurred synchronously periodic deformation cycles. In this case, the fixed optical fiber and microscope would observe and record the structural color changes of the hydrogel substrates in real time (Figure 5c). It could be found that the edge of the hydrogel experienced a wavelength shift (ranging from red to emerald green) under the actuation of cardiomyocytes. These features of the biohybrid microfluidic system laid the foundation for biomedical applications such as drug screening.

When pumping the isoproterenol‐dosed medium into the microfluidic chip, the contraction of cardiomyocytes would be accelerated and strengthened by a drug additive, which would be synchronously reflected on the structural color shifts. Specifically, the edge of the hydrogel exhibited an enhanced blue‐shift from red to blue with isoproterenol stimulation (Figure 5d; Movie S2, Supporting Information), compared to the normal cultured one. The detailed wavelength shifts were recorded by the spectrometer and further analyzed. It was found that the optical shift value of structural color hydrogel substrate stabilized near 60 nm when cardiomyocytes were cultured in a normal medium. With the addition of isoproterenol increasing, the shift value correspondingly increased up to ≈130 nm when the concentration of isoproterenol reached 10^–5^ m (Figure 5e). Besides the shift reflection of contraction force, the deformation frequency is also closely related to the cardiomyocytes beating frequency. The statistic graph demonstrated the cardiac‐driven deformation change, both in wavelength shift and shifting frequency, under different isoproterenol conditions (Figure 5f). More importantly, the wavelength shift values could be translated into stress signals for mechanical sensing of cardiomyocytes (Figure S9, Supporting Information), according to the standardized stress‐bending angle and wavelength‐bending angle curves (Figures S2 and S4, Supporting Information). Compared with existing detection platforms, our method greatly simplified the measurement and analysis procedures, while its sensitivity remained to be improved to meet clinical demands. All these outcomes revealed that the SACNT‐integrated structural color hydrogel could act as an essential heart‐on‐a‐chip visualized element and exert value in a cardiac sensing and drug evaluation.

## 3 Conclusion

In conclusion, we have exploited a novel electroconductive and anisotropic structural color hydrogel by simply coating and polymerizing non‐close‐packed colloidal arrays on SACNTs for visualized and accurate heart‐on‐a‐chip construction. The substrate, composing surface‐charged silica nanoparticles and hybrid hydrogel, was quickly and simply fabricated and demonstrated angle‐dependent optical features from hydrogel network‐locked silica nanoparticles that assembled into the non‐close‐packed microstructure. Benefiting from the biocompatibility of GelMA and conductive SACNTs of the composite hydrogel, the cultured cardiomyocytes were endowed with high cellular orientation and enhanced signal conduction, which facilitated their synchronous beating performance. Under the actuation of contraction and relaxation of cardiomyocytes, the electroconductive structural color hydrogel underwent a periodic deformation, thus contributing to the reversible structural color variation. When composited into the microfluidic chip, the optical variation of the hydrogel deformation resulting from cell actuation would across the transparent PDMS, and thus achieve the visualized cardiac sensing. Based on these features, it has been demonstrated that the derived heart‐on‐chips system was suitable for isoproterenol evaluation. Compared with the existing detection platforms such as microscopy techniques, impedance technology, and even heart‐on‐chips,^[^ ^15^, ^16^, ^23^ ^]^ our structural color‐based system not only gets rid of expensive detection equipment and complex calculations, but also simplifies the fabrication procedures and accurately imitates cardiac physiology, thus promoting the process of organs‐on‐chips toward clinical research. In the future, our effort would focus on the standardization of microfluidic chips and expanding their medical values. We conceive that the fast fabricated electroconductive structural color hydrogel had broad prospects in heart‐on‐a‐chip construction and biomedical applications such as drug screening.

## 4 Experimental Section

### Materials

Negatively charged silica nanoparticles and GelMA were self‐synthesized in the laboratory by the Stöber method according to previous studies.^[^ ^41^ ^]^ Gelatin and methacrylic anhydride that utilized for GelMA synthesis, AAm, *N,N*′‐methylenebis(acrylamide), PEGDA (Mw = 700), 2‐Hydroxy‐2‐methyl‐1‐phenyl‐1‐propanone, trypsin, pancreatin, 5‐bromo‐2‐deoxyuridine and isoproterenol were obtained from Sigma–Aldrich (St. Louis, MO). Cellulose dialysis membranes acquired from Yuanye Biotechnology Corporation (China) were employed for GelMA purification. In the biocompatibility test, 3‐(4,5‐dimethyl‐2‐thiazolyl)‐2, 5‐diphenyl‐2‐H‐tetrazolium bromide was from J&K Scientific Ltd. and dimethyl sulfoxide was derived from Macklin Biochemical Co., Ltd, respectively. SACNTs were achieved from the Beijing Funat Innovation Technology Corporation of China. Dulbecco's Modified Eagle Medium/Nutrient Mixture F‐12, Hanks’ Balanced Salt Solution, fetal bovine serum, cellular dyes including Alexa Fluor 488 Phalloidin, and 4′,6‐diamidino‐2‐phenylindole were all acquired from Life Technologies (USA). Worthington provided collagenase type 2, while Gibco (USA) supplied penicillin‐streptomycin and phosphate‐buffered saline solution.

### Preparation of Electroconductive Structural Color Hydrogel

After centrifugation, the purified silica nanoparticles (≈150 nm) were mixed into PEGDA hybrid AAm solution with different doping ratios, where ion exchange resin was added to ensure the brilliant structural color. In this system, the structural color of the composite solution could be adjusted by tuning the concentration of nanoparticles. To enhance the biocompatibility of the substrate, a thin layer of GelMA hydrogel (15% w/v, ≈50 µm) was covered on the SACNT layer along their direction for better cardiomyocyte adhesion. After that, the SACNTs were further coated by the pregel solution of silica nanoparticles. After UV exposure (365 nm) at a distance of 10 cm for 20 s and gentle removal of the glass slide, the freestanding electroconductive structural color hydrogel was obtained.

### Extraction and Cultivation of Primary Cardiomyocytes

All animal experiments were approved by the Animal Ethics Committee of Southeast University (No. 20 180 326 003) and were conducted in compliance with the Regulations for the Administration of Affairs Concerning Experimental Animals of China. The cardiomyocytes were achieved from neonatal Sprague–Dawley rats (1–2 days) provided by comparative medicine of Jinling Hospital (Nanjing, China). The extraction of the cardiomyocytes was according to the published protocol.^[^ ^15^, ^17^ ^]^ Briefly, the cardiac tissues were extracted from rats, cut into pieces, and digested into cardiomyocytes by Hanks’ Balanced Salt Solution containing trypsin, pancreatin and collagenase type 2. After centrifugation and purification, the cardiomyocytes were resuspended and supplied with Dulbecco's Modified Eagle Medium/Nutrient Mixture F‐12 solution containing 20% fetal bovine serum and 0.1 × 10^‐3^ m 5‐bromo‐2‐deoxyuridine.

### Cardiomyocyte Staining

To characterize the cellular morphology of cardiomyocytes on the electroconductive structural hydrogel, the biohybrid substrates were successively immersed in 4% paraformaldehyde solution for 30 min and 0.25% Triton X‐100 dilution for 20 min. Then, the cardiomyocytes were stained by Alexa Fluor 488 Phalloidin diluent (1:400) for 20 min at 4 ℃, and DAPI diluent (1:1000) for a minute. After each step, the biohybrid substrates was rinsed by PBS solution for three times. Finally, the cellular morphology could be observed by a fluorescent microscope.

### Construction and Operation of Microfluidics

The PDMS microfluidic chips with specific channel and chamber designs were obtained by the template molding method. In this study, the microfluidic chip was designed with S‐shaped mixing channels for uniform injection of culture medium and drug solutions, together with four micro‐pillars inside the chamber to fix the structural color hydrogels to avoid unexpected displacement. The structural color hydrogel seeded with cardiomyocytes was carefully transferred into the plasma‐treated PDMS chamber reserved with culture medium, with its tail end located at the micro‐pillar position for fixation. Subsequently, the PDMS device was bonded with a glass slide to form the desired microfluidic chip. For pumping solutions into the microfluidic chip, its inlet and outlet were connected to syringe pumps (Harvard PHD 2000 series) by polyethylene tubes.

### Characterization

The microscopic structures of the structural color hydrogels were magnified and scanned by a scanning electron microscope (Hitachi S‐3000N). A fiber‐optic spectrometer (Ocean Optics, USB2000‐FLG) was employed to measure the reflection spectra and test the optical properties of the functional hydrogel. As for the electrical performance measurement, a digital multimeter (KEITHLEY, USA) was utilized to record the resistance changes of the hydrogel during the bending process. The relationship between bending angle and stress was measured by MicroTester G2 (CellScale Biomaterials Testing, Waterloo, Canada) under the bending test mode. Fluorescent images of cardiomyocytes were obtained by a Zeiss LSM700 laser‐scanning microscope (Zeiss, Heidenheim, Germany). Optical images were derived by a CCD camera (Media Cybernetics Evolution MP5.0) equipped microscope (Olympus BX51). The mechanical properties were tested through a universal testing machine (Suns, UMT2000). The MTT assay results were scanned by a microplate reader whose model was SYNERGY|HTX.

### Statistical Analysis

Statistical data in this study were presented as mean ± SD. *p*‐Values were analyzed by one‐way analysis of variance flowed by the least significant difference test (IBM SPSS Statistics software). The significance level was indicated as *p < 0.05, **p < 0.01, ***p < 0.001. Sample sizes (*n*) were respectively indicated in figure legends.

## Conflict of Interest

The authors declare no conflict of interest.

## Supporting information

Supporting Information

Supplemental Movie 1

Supplemental Movie 2

## Data Availability

The data that support the findings of this study are available in the supplementary material of this article.
