# Supplementary material for: Electroconductive and Anisotropic Structural Color Hydrogels for Visual Heart‐on‐a‐Chip Construction
Source: Adv Sci (Weinh). 2022 Mar 28;9(16):2105777. doi: 10.1002/advs.202105777 (PMC9165491; doi:10.1002/advs.202105777)
Supplement: Supplementary file 1 — Supporting Information [file ADVS-9-2105777-s002.pdf]

Supporting Information

Electroconductive and anisotropic structural color hydrogels for visual heart-on-a-chip construction

*Lingyu Sun, Zhuoyue Chen, Dongyu Xu, Yuanjin Zhao**

Author contributions

Y.J.Z. conceived the idea and designed the experiment; Z.Y.C conducted experiments; Z.Y.C and L.Y.S analyzed the data; L.Y.S and Y.J.Z. wrote the manuscript; D.Y.X. contributed to the scientific discussion of the article.

Supporting Figures:

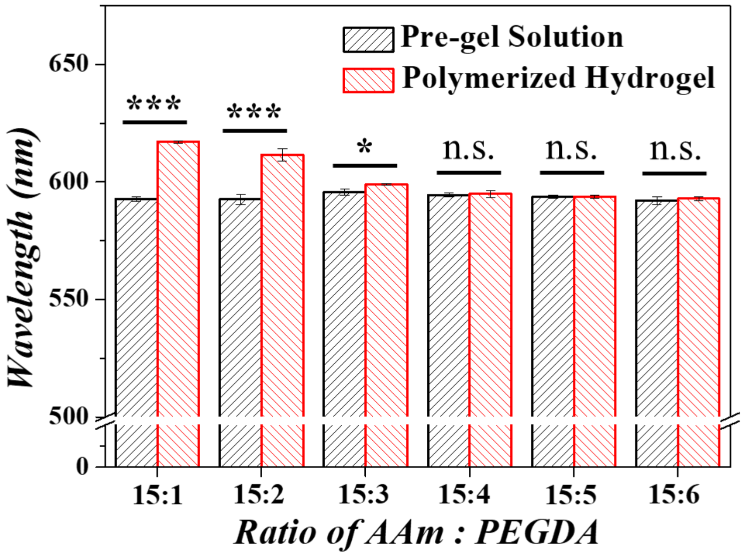

Figure S1. The Relationship between wavelength shift and the ratio of AAm : PEGDA before and after polymerization (n=3). n.s. represents no significance. * $p < 0.05$, *** $p < 0.001$.

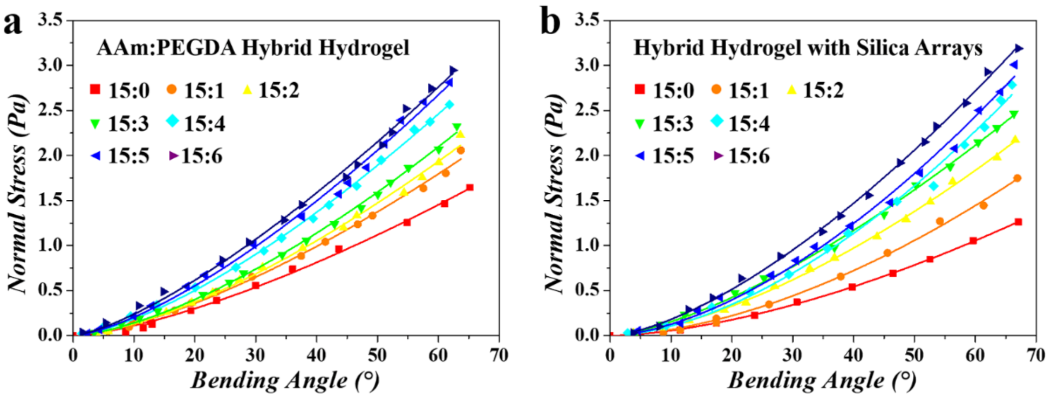

Figure S2. (a, b) Relationship between the stress and bending angle of (a) AAm : PEGDA hybrid hydrogel and (b) AAm : PEGDA hybrid hydrogel with silica arrays.

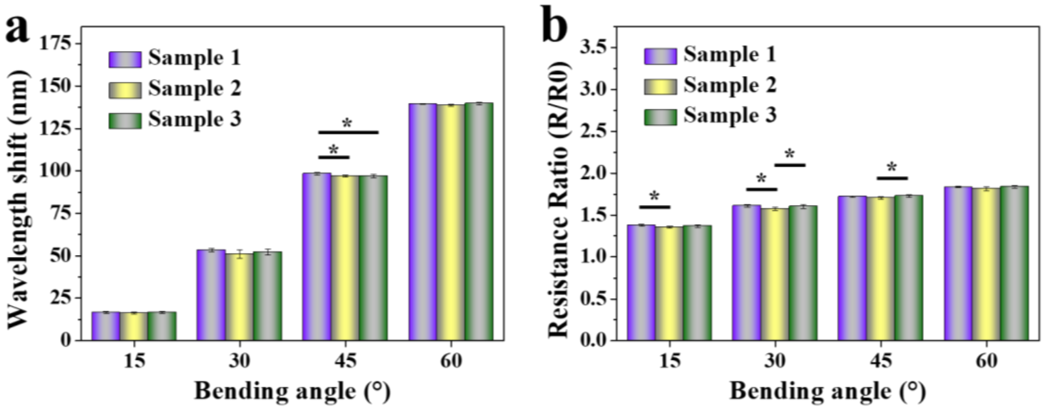

Figure S3. (a, b) The reproducibility testing of the electroconductive substrates showing the relationship between (a) wavelength shift and (b) resistance change with different bending degrees. * $p < 0.05$ (n=5 for each sample).

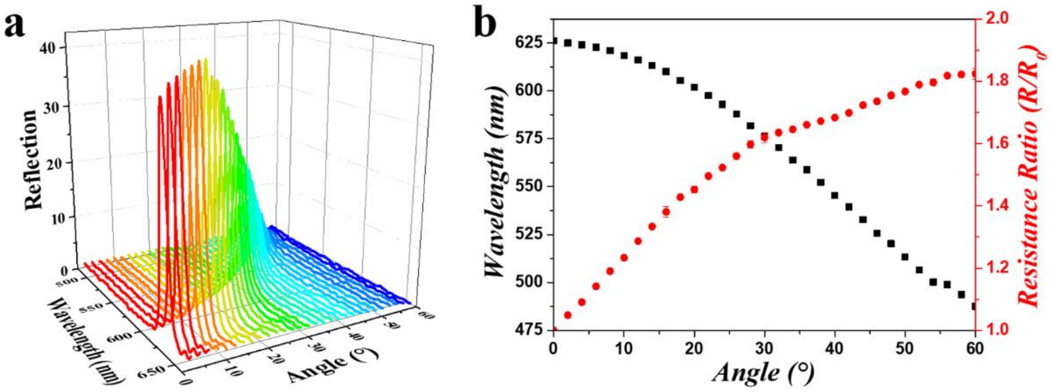

Figure S4. (a) Relationship between the reflection peaks and bending angles. (b) Statistic graph showing the relationship of wavelengths (black) and relative resistance change (red) with bending angles.

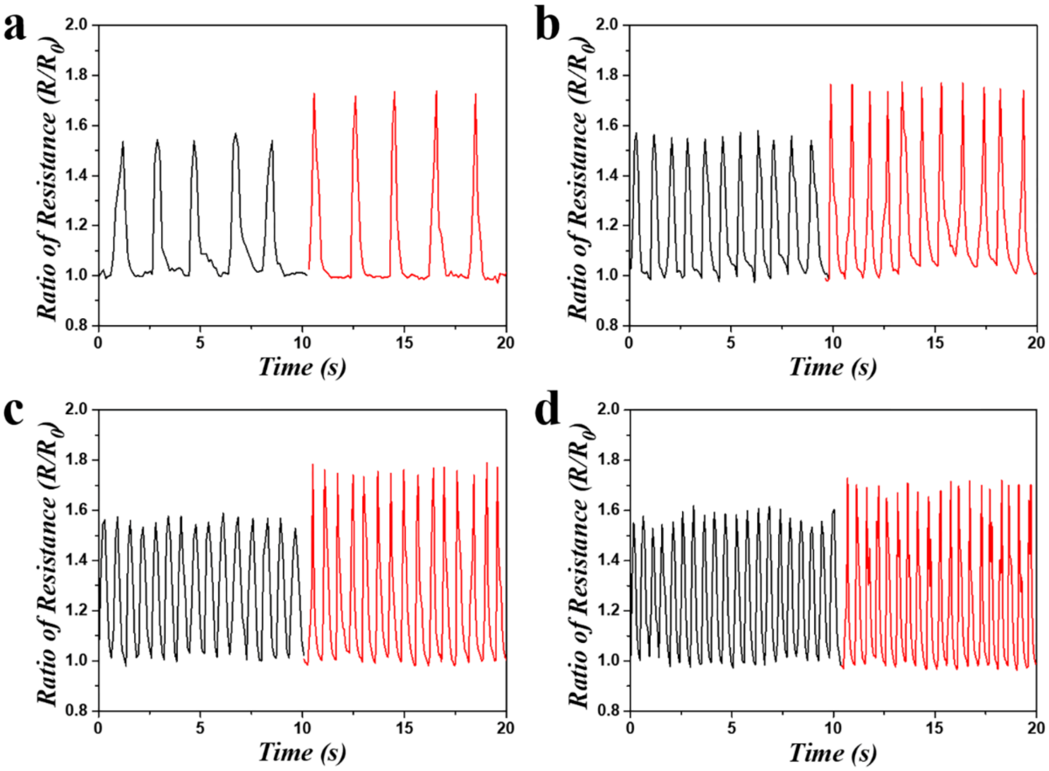

Figure S5. (a-d) Cycled tests of the relative resistance change under different bending frequencies: (a) 0.5 Hz, (b) 1 Hz, (c) 1.5 Hz and (d) 2 Hz.

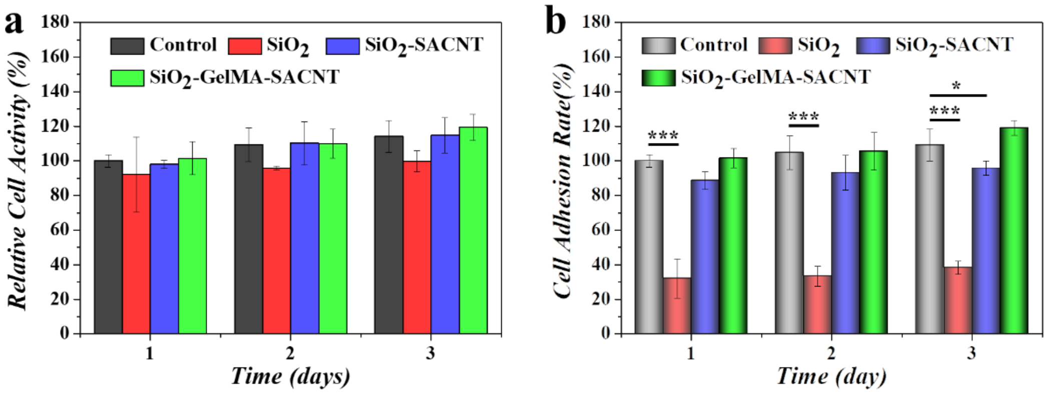

Figure S6. (a) Biocompatibility test of different groups for three days. There is no signifigance between control group and other groups (n=3). (b) Cellular adhesion test of different groups for three days. * $p < 0.05$ and *** $p < 0.001$ when compared with control group (n=3).

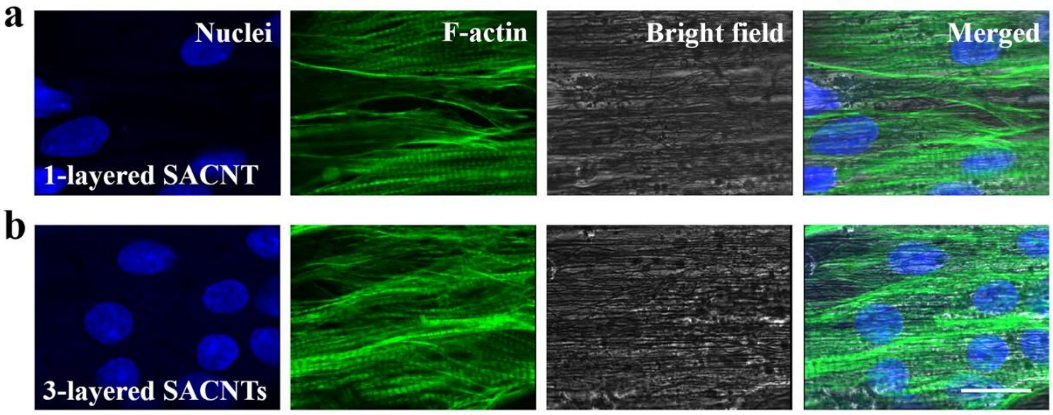

Figure S7. (a, b) Fluorescent images of the cardiomyocyte alignment on substrates with different layers of SACNTs. Scale bar is 20 μm.

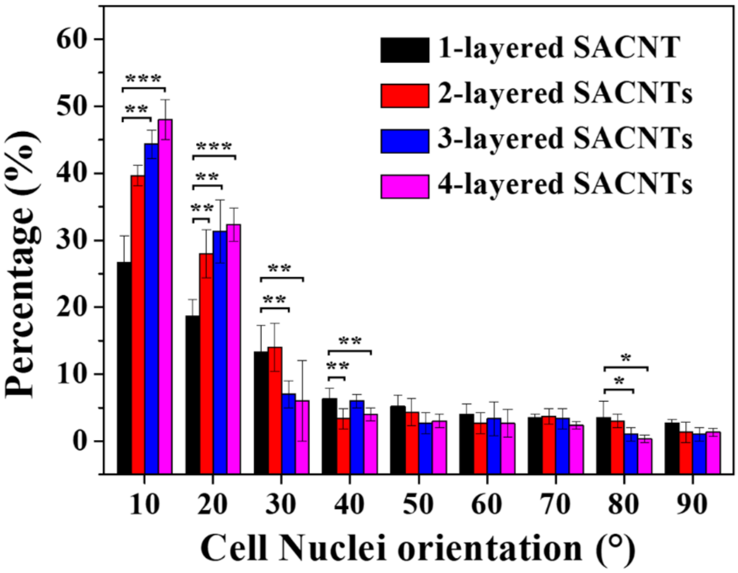

Figure S8. Statistic graph showing the cell nuclei orientation of the cardiomyocytes seeded on substrates with different layers of SACNTs. * $p < 0.05$, ** $p < 0.01$, *** $p < 0.001$ (n=3).

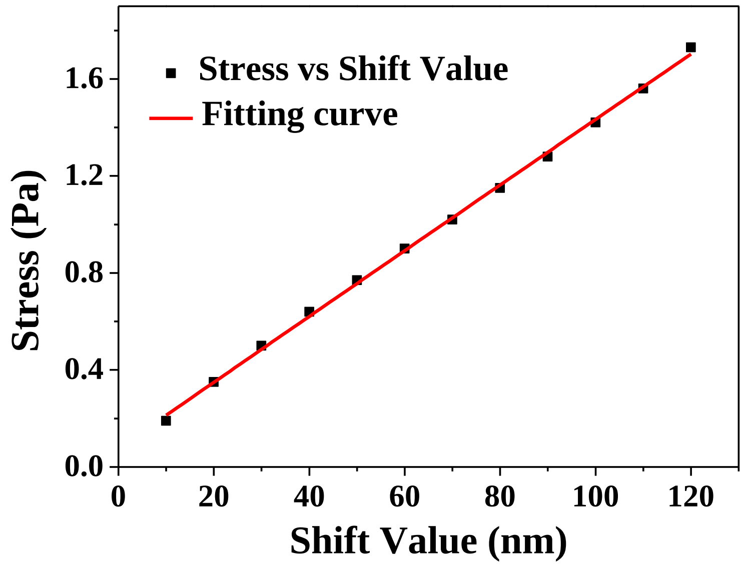

Figure S9. Relationship between wavelength shift value and the stress.

Supporting Movies:

Movie S1. The beating of cardiomyocytes on substrate without SACNT integration (first half) and on SACNT-integrated substrate (second half).

Movie S2. The bending process of the electroconductive structural color hydrogel in normal culture medium (first half) and under isoproterenol stimulation (second half).
